# Supplementary material for: Nanobead-based single-molecule pulldown for single cells
Source: Heliyon. 2023 Nov 14;9(11):e22306. doi: 10.1016/j.heliyon.2023.e22306 (PMC10679481; doi:10.1016/j.heliyon.2023.e22306)
Supplement: Multimedia component 1 [file mmc1.pdf]

Supporting Information

Qirui Zhao, Yusheng Shen, Xiaofen Li, Yulin Li, Fang Tian, Xiaojie Yu, Zhengzhao Liu, Rongbiao Tong, Hyokeun Park, Levent Yobas, Pingbo Huang*

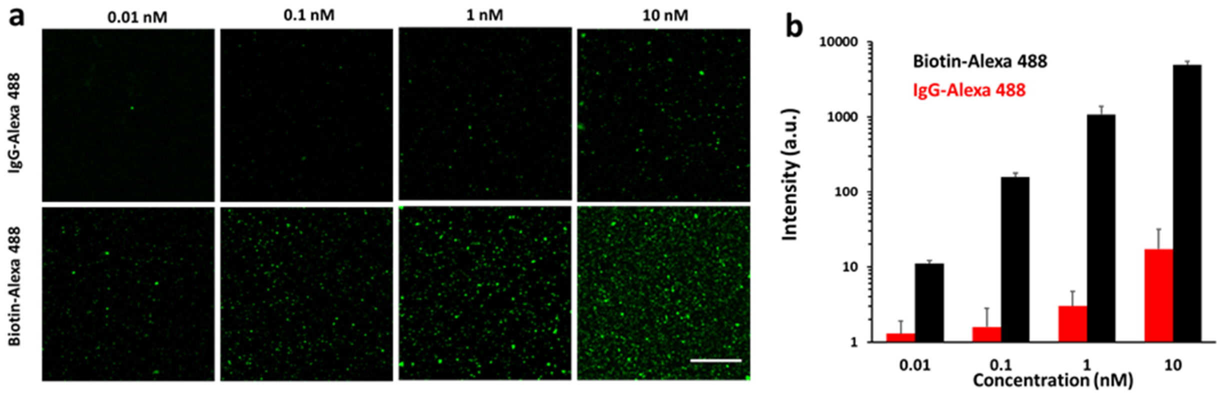

Supplementary Figure 1. Detection sensitivity of streptavidin-coated magnetic nanobeads for biotin-Alexa 488. a) Various concentrations (0.01–10 nM) of IgG-Alexa 488 (upper) or biotin-Alexa 488 (lower) pulled down by streptavidin-coated nanobeads after incubation for 10 min. The panel shows 30 × 30 μm imaging areas selected from a glass slide. Scale bar: 10 μm. **b)** Average signal intensity of imaging areas in **(a)** plus two similar imaging areas in the same experiment. The nanobeads detected biotin-Alexa 488 at a concentration as low as 10 pM with an extremely high S/B ratio (>9 relative to IgG-Alexa 488; Y-axis is in the logarithmic scale).

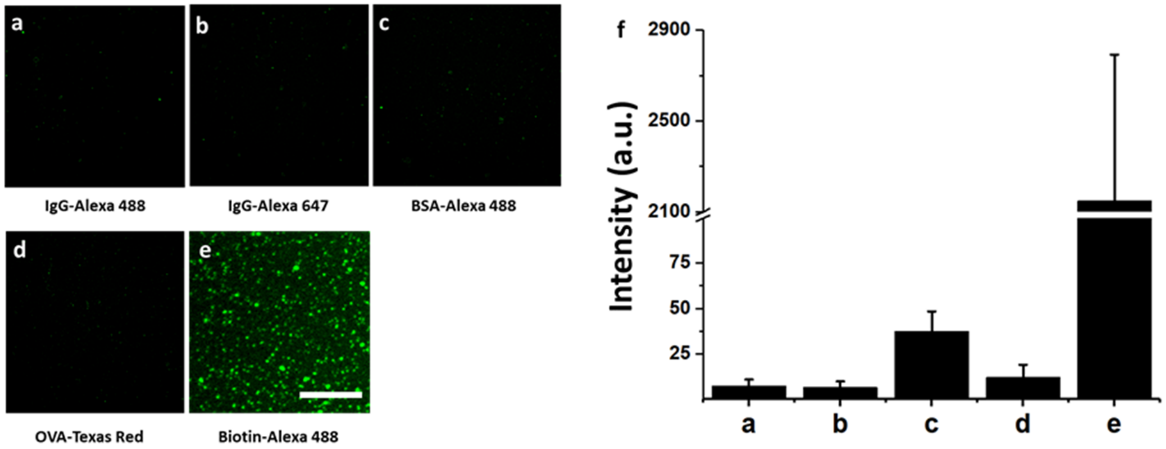

Supplementary Figure 2. Minimal nonspecific binding of streptavidin-coated magnetic nanobeads. a-e) Binding of 200 nM IgG-Alexa 488 **(a)**, IgG-Alexa 647 **(b)**, BSA-Alexa 488 **(c)**, or OVA-Texas Red **(d)** or of 100 nM biotin-Alexa 488 **(e)** to streptavidin-coated magnetic nanobeads after incubation for 10 min. The panels show 30 × 30 μm imaging areas. Scale bar: 10 μm. **f)** Average signal intensity of imaging areas in **(a-e)** plus two similar imaging areas in the same experiment.

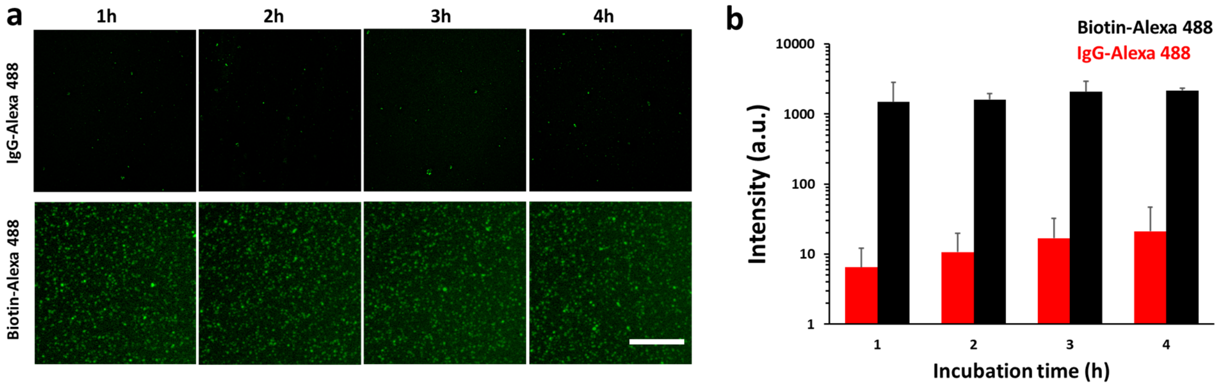

Supplementary Figure 3. Time course of nonspecific binding of IgG-Alexa 488 to magnetic nanobeads. a) Nonspecific binding of IgG-Alexa 488 to streptavidin-coated magnetic nanobeads after different incubation times. Biotin-Alexa 488: positive control. Scale bar: 10 μm. **b)** Average signal intensity of imaging areas in **(a)** plus two similar imaging areas in the same experiment. Incubation time with 1st or 2nd antibody was considerably shorter (10–30 min) in other experiments (see "Materials and Methods").

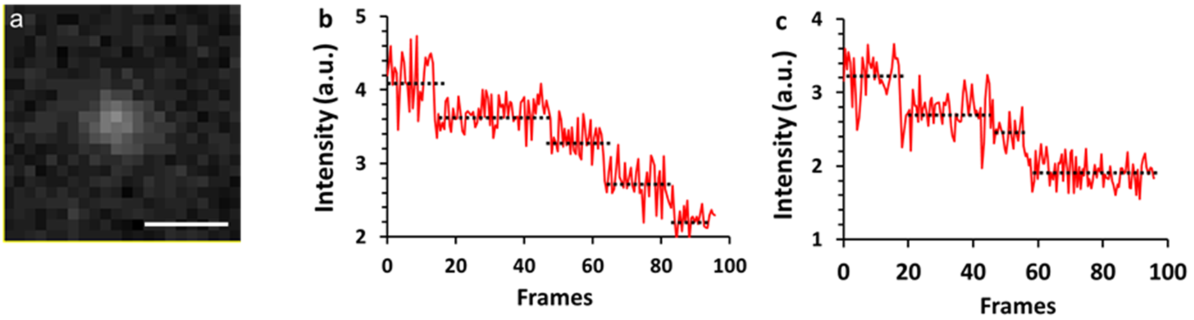

Supplementary Figure 4. a) One PKA complex comprising PKA-R-GFP and PKA-C-GFP was pulled down on a coverslip and subject to photobleaching. Scale bar: 0.5 μm. **b-c)** PKA complex showing 4 **(b)** or 3 **(c)** bleaching steps. Photobleaching was recorded at 3 frames/s.

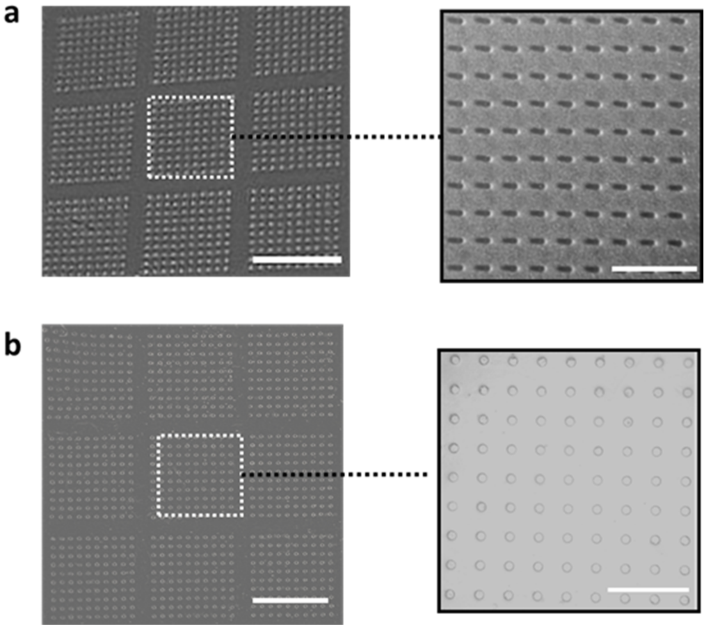

Supplementary Figure 5. Fabrication of microwell substrates. a) Microposts were fabricated on a silicon wafer by using standard soft-lithography methods, and the features were arranged in a square configuration containing 9 blocks of 10 × 10 micropillars (30 µm in diameter and 70 µm in height). **b)** PDMS gel with the corresponding features of the wafer shown in **(a)**. Scale bars: 1.5 mm in left images, 400 µm in right images.

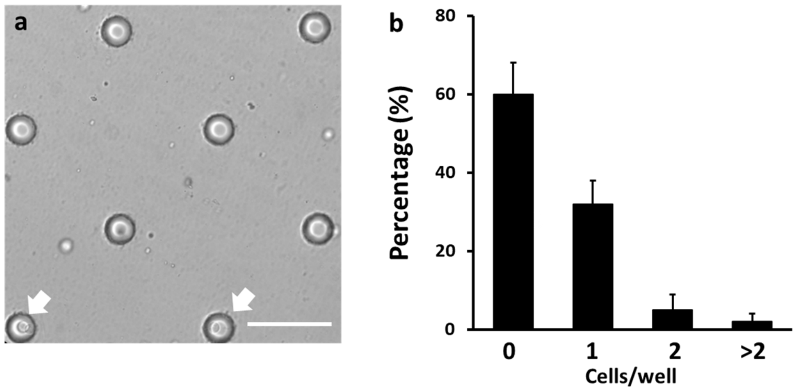

Supplementary Figure 6. Number of cells per microwell. To the surface of a microwell chip, 300 µL of suspended cells in PBS (~106 cells/mL) was applied (see additional details in "Materials and Methods"). a) Microwells containing trapped cells in the view field (white arrows). Scale bar: 100 µm. **b)** Distribution of cell numbers per well in a chip (n = 3 independent biological replicates, each representing the count of 100 randomly selected wells); ~40% of total microwells were

occupied by cells, and ~85% of cell-occupied microwells contained single cells (this ratio is expected to increase when cell suspensions are applied at higher dilutions).

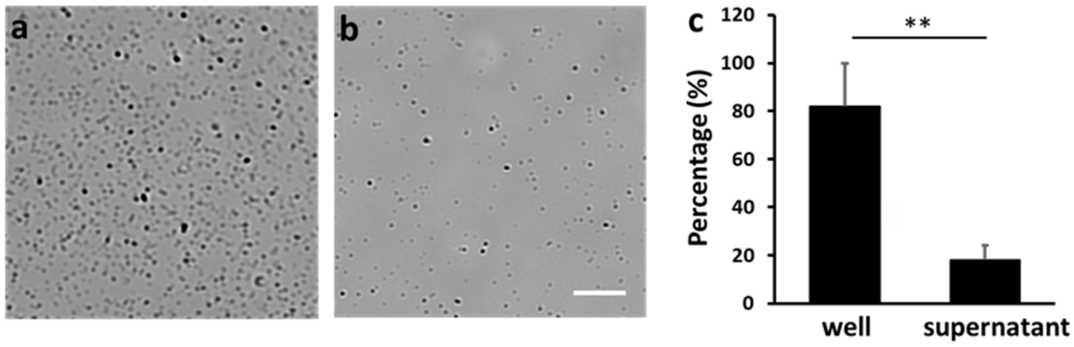

Supplementary Figure 7. Fraction of magnetic nanobeads dragged down by a magnet. a) To a microwell array chip, 10^9 magnetic nanobeads were added (in 200 µL of PBS) and pulled down into microwells after dragging for 3 min with a magnet, and the nanobeads absorbed onto the chip surface and microwell bottom were counted. **b)** Part of the supernatant on the top of the chip was collected and pipetted onto a coverslip to determine the number of magnetic nanobeads in the supernatant. Scale bar: 5 µm. **c)** Statistical data for **(a)** and **(b)**.

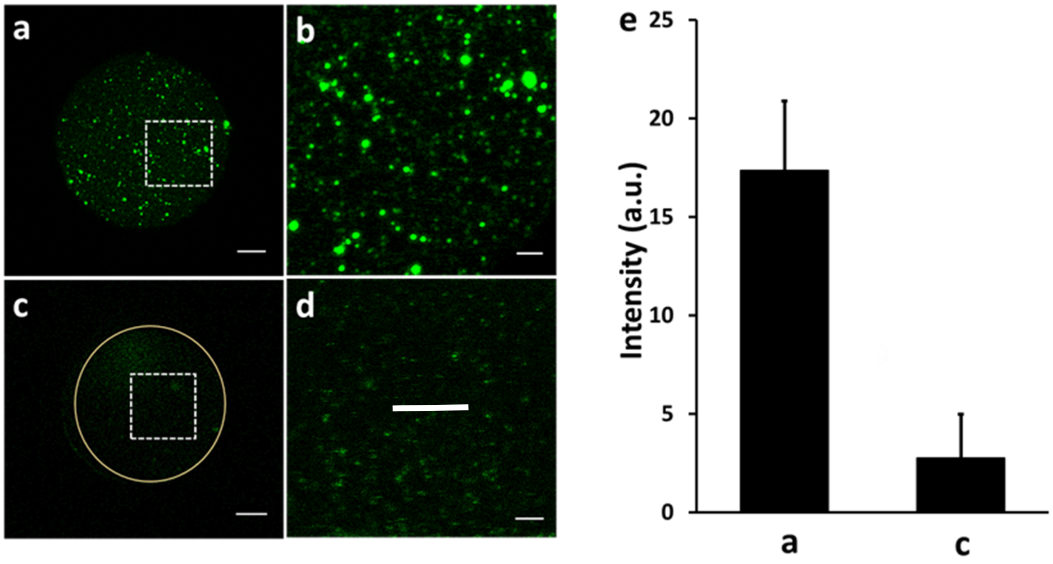

Supplementary Figure 8. a-d) Transmembrane protein ANO1-GFP from a single HEK293T cell was pulled down by using magnetic nanobeads coated with **(a)** or without **(c)** anti-GFP after cell lysis in a microwell; **(b)** and **(d)** show magnified images of boxed areas in **(a)** and **(c)**, respectively.

Scale bars: 5 μm in **(a)** and **(c)**, 2 μm in **(b)** and **(d)**. **e)** Summary data for **(a)** and **(c)**. S/B ratio = 6.4; n = 3 images from the same experiment.

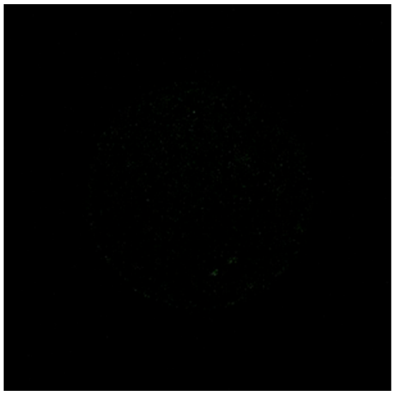

Supplementary Video 1. GFP in a single cell trapped in a microwell (30 μm in diameter) was pulled down using magnetic beads after adding lysis buffer.
